# Associations Between Blood Concentrations of Sex Hormones and Physical Function in Community-Dwelling Older Women: A Prospective Cohort Study

**DOI:** 10.1093/gerona/glad287

**Published:** 2023-12-29

**Authors:** Yuanyuan Wang, Rakibul M Islam, Sultana Monira Hussain, John J McNeil, Susan R Davis

**Affiliations:** School of Public Health and Preventive Medicine, Monash University, Melbourne, Victoria, Australia; School of Public Health and Preventive Medicine, Monash University, Melbourne, Victoria, Australia; School of Public Health and Preventive Medicine, Monash University, Melbourne, Victoria, Australia; School of Public Health and Preventive Medicine, Monash University, Melbourne, Victoria, Australia; School of Public Health and Preventive Medicine, Monash University, Melbourne, Victoria, Australia; Department of Endocrinology and Diabetes, Alfred Health, Melbourne, Victoria, Australia; (Medical Sciences Section)

**Keywords:** dehydroepiandrosterone, grip strength, estrone, testosterone, women

## Abstract

**Background:**

Blood concentrations of testosterone and estrone tend to increase in women aged ≥70 years, whereas concentrations of their precursor hormone dehydroepiandrosterone decline. It is unknown whether these changes influence physical function. We investigated whether concentrations of these hormones were associated with grip strength and self-reported physical function in community-dwelling older women.

**Methods:**

A total of 9 179 Australian women, aged ≥70 years, were recruited to the ASPirin in Reducing Events in the Elderly (ASPREE) trial. Sex hormones were measured in Sex Hormones in Older Women, an ASPREE substudy, by liquid chromatography–tandem mass spectrometry in 6 358 women. The associations between hormone concentrations and physical function (handgrip strength and self-reported physical function assessed by SF-12v2 physical component summary [PCS]) were examined using multiple linear regression.

**Results:**

The median age of the 5,447 participants was 74.0 (interquartile range 71.7–77.6) years. Testosterone concentrations above the lowest quartile were associated with less decline in grip strength (mean –1.39 [95% CI –1.54 to –1.24] vs –1.75 [–2.00 to –1.50] kg, *p* = .02), and dehydroepiandrosterone concentrations above the lowest quartile were associated with less decline in grip strength (–1.39 [–1.54 to –1.25] vs –1.82 [–2.11 to –1.55] kg, *p* = .007) and PCS scores (–1.49 [–1.80 to –1.17] vs –2.33 [–2.93 to –1.72], *p* = .02) over 4 years, compared with those in the respective lowest quartile.

**Conclusions:**

Low endogenous concentrations of testosterone and dehydroepiandrosterone were associated the greatest likelihood of physical function decline in community-based women aged ≥70 years. Further studies are warranted to determine whether testosterone and dehydroepiandrosterone therapy prevent functional decline in this at-risk group using sensitive measures of muscle strength and performance.

Key health goals for older people include maintaining functional independence, reducing premature disability, and optimizing quality of life. Maintenance of adequate physical function and preservation of mobility are critical for maintaining independence for healthy aging (1). Greater understanding of the modifiable factors associated with loss of physical function in older adults may help identify the at-risk individuals most likely to benefit from interventions to slow functional decline with age.

The Sex Hormones in Older Women (SHOW) Study of women aged 70–94 years (2) was a substudy of the ASPirin in Reducing Events in the Elderly (ASPREE) trial (3). The SHOW Study affirmed that as suggested in prior studies (4,5), blood concentrations of testosterone and estrone increase from the age of 70 years in women, whereas concentrations of their precursor hormone, dehydroepiandrosterone (DHEA), concurrently decline. Why this occurs and whether higher testosterone and estrone confer a survival advantage for older women remain uncertain. Our systematic review of the published literature did not support an association between endogenous testosterone concentrations and muscle mass, strength, or performance in postmenopausal women (6). However, the included studies were limited by the use of immunoassays that lack precision for measuring testosterone in postmenopausal women, and nonexclusion of women taking medications that influence sex hormone concentrations (6). Low blood concentrations of DHEA sulfate (DHEAS), a circulating reservoir for DHEA tissue production, have been associated with weaker grip strength and a decline in functional performance in community-dwelling older women (7–9). To our knowledge, the association between blood concentrations of estrone, the main circulating estrogen in women after menopause (10), and physical function in older women has not been reported. Longitudinal data for the associations between endogenous concentrations of sex hormones measured with precision and the change in physical function in older women are scant.

Physical function can be evaluated by measures of muscle strength and self-reported day-to-day function. Grip strength is an established indicator of upper limb muscular strength and body function and has been suggested as a biomarker of aging (11,12). Weak grip strength is predictive of increased risks of falls and fractures, disability, dementia, and all-cause mortality (13–16). Physical health-related quality of life assessed by the Medical Outcomes Study Short Form 12 Health Survey (SF-12) provides information about physical health symptoms and functioning and overall health status, and has been shown to effectively predict adverse health outcomes, including cardiovascular disease, cognitive decline, dementia, and mortality (17–19). The aim of the present study was to evaluate the associations between blood concentrations of sex hormones and conventional measures of physical function in the SHOW Study participants who were community-dwelling, initially healthy, older women, with prospective data collected over 4 years.

## Method

### Study Design and Participants

The current study analyzed data from the ASPREE clinical trial (3) and the ASPREE substudy, SHOW (2). ASPREE recruited participants between March 10, 2010, and December 31, 2014 (3). In brief, ASPREE was a randomized placebo-controlled trial to determine whether 100 mg/day aspirin extended disability-free survival over 5 years in community-dwelling older adults (3,20). Individuals were excluded if they had a history of cardiovascular events, dementia, or a score of <78 on Modified Mini-Mental State Examination (21), physical disability (major difficulty with independently performing any one of 6 basic activities of daily living) (22), clinically significant anemia, uncontrolled hypertension (systolic blood pressure ≥180 mmHg or diastolic blood pressure ≥105 mmHg), or a life expectancy of <5 years. This resulted in a relatively healthy, independently-living cohort at trial enrollment. Among the 16 703 Australian participants of the ASPREE trial (all aged ≥70 years at enrollment), 9 179 were women, of whom 6 392 (69.6%) provided biobank samples for the SHOW Study. The ASPREE trial was registered with the International Standard Randomized Controlled Trial Number Register (ISRCTN83772183) and clinicaltrials.gov (NCT01038583), and approved by the ethics committee at each participating center. All participants provided written informed consent. Participants contributing biospecimens to the ASPREE Healthy Ageing Biobank provided written informed consent. The SHOW Study was approved by the Monash University Human Research Ethics Committee (CF16/10 - 2016000001) and the Alfred Hospital Ethics Committee (616/15).

### Measurement of Sex Hormones

Nonfasting blood samples were collected at or within 12 months of participant recruitment, and plasma aliquots were stored under nitrogen vapor. In the SHOW Study, sex hormones were measured by liquid chromatography–tandem mass spectrometry (LCMS) in a single plasma sample at the ANZAC Research Institute (Sydney, NSW, Australia) (2). Testosterone, DHEA, and estrone were quantified within a single run without derivatization, as previously described (2,23,24). Deuterated isotopes were d3-testosterone, d2-DHEA, and d4-estrone. All steroid standards and internal standards were from the National Measurement Institute (Sydney, NSW, Australia), except for those for d4-estrone (Steraloids; Newport, RI) and estrone (Cerilliant; Round Rock, TX). The LCMS was performed on an API-5000 triple-quadrupole mass spectrometer (Applied Biosystems; Foster City, CA), equipped with an atmospheric pressure photoionization source that operated in both positive and negative ion modes. Over 3 quality control concentrations for measurements, the limit of detection was 0.035 nmol/L for testosterone, 0.070 nmol/L for DHEA, and 3.700 pmol/L for estrone; the limit of quantification was 0.09 nmol/L for testosterone, 0.17 nmol/L for DHEA, and 11.00 pmol/L for estrone. The within-run range of coefficient of variation was 2.0% for testosterone, 3.0%–6.0% for DHEA, and 4.7% for estrone, and the between-run range of coefficient of variation was 3.9%–6.5% for testosterone, 8.0%–12.0% for DHEA, and 4.6%–7.5% for estrone (25).

As part of the SHOW Study, of the 2 688 women who had blood biobank samples 3 years post-recruitment and who were not using anti-estrogen, hormone therapy, or anti-androgen, a subsample of 450 women was randomly selected across the age range to determine whether sex hormones changed with age (26). By chance, 451 paired samples were sent for analysis of which there were 3 participants with no sample and 2 participants with data transcription errors. We further excluded another 46 women on excluded medications, resulting in 400 paired samples of SHOW participants available for an exploratory analysis. All paired samples were measured in the same assay run (26). Of the 400 participants, 392 had physical function measured at ASPREE study entry and were included in the exploratory analyses of the current study.

### Assessment of Physical Function

At the baseline visit of the ASPREE trial, grip strength and self-reported physical function were assessed using validated methods as described and reported previously (27). These data were also collected at the 4-year follow-up visit. Grip strength was measured in kilograms using a hand-held isometric dynamometer (Jamar; JLW Instruments). Participants were in a seated position with the arm at the side of the body, wrist neutral, and elbow flexed to 90°. Grip strength was measured with 3 trials with a 15–20 second rest in between on each hand. The mean of the 3 measures for each hand was taken as the grip strength of that hand, and the grip strength of the self-reported dominant hand was used in the analysis. A grip strength of lower than 16 kg was classified as weak grip strength (28).

Health-related quality of life was assessed using the SF-12 Version 2 (SF-12v2), consisting of 12 items measuring 8 health domains to assess physical and mental health (29). The scores of these domains were weighted and summarized into physical component summary (PCS) score, ranging 0–100 with higher values indicating better health status (30). Self-reported physical function was assessed using the PCS score. A poor physical function was defined by a SF-12 PCS score of ≤50 (31).

### Covariates

At baseline, weight and height were measured, with body mass index (BMI) calculated from weight (kg)/height (m)^2^. Date of birth, smoking, and alcohol consumption were collected using questionnaires as previously described (20). Diabetes was defined as a fasting plasma glucose concentration of ≥7 mmol/L, on treatment for diabetes, or self-report. Chronic kidney disease was defined as an estimated glomerular filtration rate of <60 mL/min/1.73 m².

### Statistical Analysis

Hormone concentrations, with skewed distributions, were expressed by median (interdecile range [IDR]) and examined by quartiles. For samples with a hormone concentration below the limit of detection, the concentration was estimated by dividing the limit of detection by √2, applied for estrone (*n* = 88), testosterone (*n* = 126), and DHEA (*n* = 57). This approach, which allows for analysis of the full data set, has been shown to be an appropriate method to minimize left censoring bias (32). Multiple linear regression was used to examine the associations between hormone concentrations and physical function (grip strength and PCS score) at baseline, adjusted for age, BMI, alcohol consumption, smoking, diabetes, and chronic kidney disease. The continuous variables of physical function were categorized into dichotomous variables, using the cut-points for the identification of clinically relevant weakness and poor physical condition (28,31). The association between hormone concentrations and the dichotomous physical function outcomes was examined using binary logistic regression, adjusted for age, BMI, alcohol consumption, smoking, diabetes, and chronic kidney disease. The change in physical function was calculated by subtracting the baseline value from the 4-year follow-up value, with negative values indicating a decline of physical function. Multiple linear regression was used to examine the association between hormone concentrations at baseline and the change in physical function over 4 years, adjusted for age, BMI, alcohol consumption, smoking, diabetes, chronic kidney disease, treatment allocation (aspirin/placebo), and respective baseline physical function measure. When baseline hormone concentrations were examined in regression models, *F* omnibus tests were performed to determine whether there were differences between the hormone concentration quartiles. Quartiles 2, 3, and 4 were combined if there were no statistically significant differences between the 3 groups.

The interaction between obesity status (BMI <30 kg/m^2^ vs BMI ≥30 kg/m^2^) or age (aged <75 years vs aged ≥75 years based on the median age) and hormone concentrations for their association with physical function was examined. Given the change in sex hormone concentrations with age in older women (2,4), we examined whether baseline hormone concentrations were associated with the changes in physical function over 4 years after controlling for the changes in hormone concentrations over 3 years from baseline, in a subgroup of participants with sex hormones measured at 3 years after baseline. We also performed exploratory analysis to examine the association between the change in hormone concentrations at 3 years post-baseline (in quartiles) and the changes in physical function over 4 years. All statistical tests were 2-sided and a *p* value of <.05 was considered statistically significant. All statistical analyses were performed using Stata MP version 17.0 (StataCorp LP, College Station, TX).

## Results

**Figure 1.**
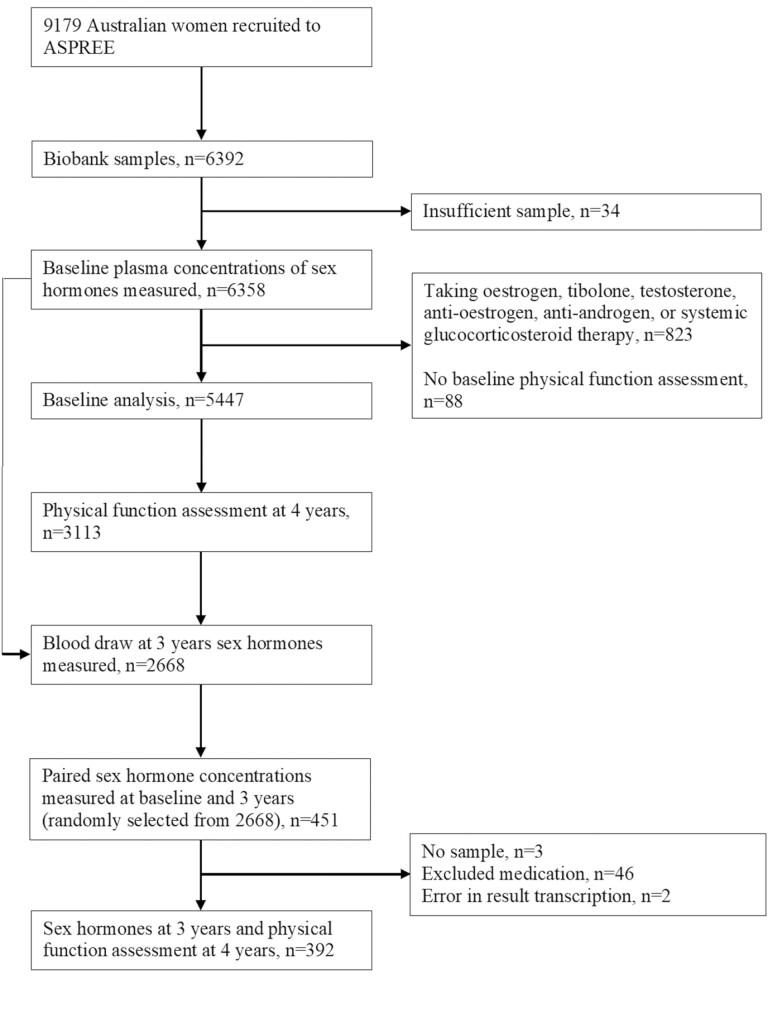
Study profile.

Of the 9 179 Australian, female ASPREE trial participants, 6 392 (69.6%) provided biobank samples for the SHOW Study, and of these 6 358 (99.5%) provided sufficient samples from which baseline plasma concentrations of sex hormones were measured. After exclusion of participants taking sex hormones, anti-estrogens, anti-androgens, or systemic glucocorticoids (*n* = 823) and those who did not have physical function assessed at study entry (*n* = 88), 5 447 participants were included in the current study (Figure 1).

Participants who provided sufficient blood samples for sex hormone measurement (*n* = 6 358) were slightly younger (median age 73.9 [interquartile range, IQR, 71.7–77.5] vs 75.0 [72.4–79.1] years), and had slightly higher grip strength (mean 21.0 [standard deviation {*SD*} 5.4] vs 20.0 [5.4] kg) and PCS score (mean 47.7 [*SD* 9.1] vs 46.9 [9.3]), compared with those who did not (*n* = 2 821). There were no differences in BMI, smoking, alcohol consumption, diabetes, and chronic kidney disease between the 2 groups.

The baseline characteristics of study participants are presented in Table 1. The 5 447 participants were aged 70.0–94.8 years, with a median age of 74.0 years (IQR 71.7–77.6). Their mean BMI was 28.1 kg/m^2^, and over 70% were overweight or had obesity. Over 4.0 (*SD* 0.1) years, 3 113 (57.2%) participants had physical function assessed at the 4-year follow-up. At baseline, these participants were less likely to have diabetes (214 [6.9%] vs 218 [9.3%]) and had higher PCS scores (48.3 [*SD* 8.8] vs 47.3 [9.5]) compared with those who did not have physical function reassessed (Supplementary Table 1).

**Table 1.** Characteristics of Study Participants at Baseline

	*N* = 5 447
Age, years	74.0 (IQR 71.7–77.6)
Body mass index, kg/m^2^	28.1 (5.1)
Body mass index categories, no (%)	
<18.5 kg/m^2^	44 (0.8)
18.5–24.9 kg/m^2^	1 537 (28.4)
25.0–29.9 kg/m^2^	2 148 (39.6)
≥30.0 kg/m^2^	1 691 (31.2)
Current/former smoker, no (%)	1 866 (34.3)
Current/former alcohol user, no (%)	4 280 (78.6)
Aspirin group, no (%)	2 726 (50.0)
Diabetes, no (%)	432 (7.9)
Chronic kidney disease, no (%)	990 (18.7)
Testosterone, nmol/L	0.38 (IDR 0.17–0.87)
Quartile 1, *n* = 1 605	0.17 (0.10–0.24)
Quartile 2, *n* = 1 411	0.31 (0.28–0.38)
Quartile 3, *n* = 1 150	0.49 (0.42–0.55)
Quartile 4, *n* = 1 281	0.80 (0.59–1.98)
Estrone, pmol/L	181.23 (IDR 85.07–343.97)
Quartile 1, *n* = 1 376	96.16 (48.08–122.05)
Quartile 2, *n* = 1 403	155.34 (133.15–177.53)
Quartile 3, *n* = 1 335	218.22 (188.63–255.20)
Quartile 4, *n* = 1 333	329.18 (277.40–477.12)
Dehydroepiandrosterone, nmol/L	2.63 (IDR 1.04–6.14)
Quartile 1, *n* = 1 393	1.14 (0.55–1.59)
Quartile 2, *n* = 1 324	2.15 (1.77–2.50)
Quartile 3, *n* = 1 379	3.22 (2.74–3.85)
Quartile 4, *n* = 1 351	5.62 (4.23–8.63)
Grip strength, kg	21.1 (5.3)
Physical component summary score (0–100)	47.9 (9.1)

*Notes*: Data are presented as median (interquartile range [IQR]/interdecile range [IDR]), mean (standard deviation), or no (%). SF = Short Form.

Sex hormone concentrations at 3 years from baseline were available for 392 (7.2%) of the 5 447 participants in the SHOW Study. Participants with paired hormone measures were older (median age 78.0 [IQR 73.6–82.2] vs 73.8 [71.6–77.2] years), had weaker grip strength (mean 20.1 [*SD* 5.6] vs 21.2 [5.3] kg), higher baseline estrone (median 203.42 [IDR 99.86–384.65] vs 177.53 [85.07–343.97] pmol/L), and lower baseline DHEA (median 2.39 [IDR 1.04–4.78] vs 2.63 [1.04–6.28] nmol/L) compared with those who did not have sex hormone measured at 3 years. Over the 3 years, the median testosterone concentration increased from 0.35 (IDR 0.14–0.83) to 0.38 (0.17–0.94) nmol/L (*p* = .02), with no significant changes in estrone or DHEA.

Baseline grip strength was first examined as a continuous variable with adjustment for age, BMI, alcohol, smoking, diabetes, and chronic kidney disease (Table 2). Statistically significant greater grip strength was seen for participants with DHEA concentrations in quartile (Q) 3 (mean 21.3 kg, 95% CI 21.0–21.6) and Q4 (21.3 kg, 21.0–21.6), and Q2 to Q4 combined (21.2 kg, 21.0–21.3) compared with those with Q1 (20.8 kg, 20.6–21.1). There was no significant association between grip strength and testosterone, and an isolated statistically significant greater grip strength for estrone concentrations in Q2 compared with Q1. When grip strength was examined as a dichotomous variable, no significant associations for any of the sex hormones were seen for weak grip strength versus not weak strength.

**Table 2.** Association Between Baseline Plasma Concentrations of Sex Hormones and Grip Strength at Baseline (*n* = 5 447) and Its Change Over 4 Years (*n* = 3 113)

	Grip Strength at Baseline (kg)^*^		Weak Grip Strength at Baseline^*^		Change in Grip Strength (kg)^†^	
	Mean (95% CI)	*p* Value	Odds Ratio (95% CI)	*p* Value	Mean (95% CI)	*p* Value
Testosterone						
Quartile 1	21.0 (20.7, 21.2)		1.00		–1.75 (–2.00, –1.50)	
Quartile 2	21.0 (20.7, 21.2)	.90	1.04 (0.85, 1.28)	.70	–1.69 (–1.94, –1.44)	.75
Quartile 3	21.2 (20.9, 21.5)	.19	0.89 (0.71, 1.11)	.30	–1.32 (–1.60, –1.05)	.02
Quartile 4	21.2 (20.9, 21.5)	.28	0.96 (0.78, 1.18)	.69	–1.13 (–1.39, –0.87)	.001
Quartiles 2–4	21.1 (21.0, 21.3)	.37	0.97 (0.82, 1.15)	.70	–1.39 (–1.54, –1.24)	.02
Estrone						
Quartile 1	20.9 (20.6, 21.2)		1.00		–1.64 (–1.93, –1.36)	
Quartile 2	21.3 (21.0, 21.6)	.04	0.85 (0.68, 1.06)	.16	–1.60 (–1.85, –1.34)	.80
Quartile 3	21.0 (20.7, 21.3)	.53	1.00 (0.80, 1.24)	.99	–1.44 (–1.70, –1.19)	.30
Quartile 4	21.1 (20.8, 21.4)	.37	0.99 (0.79, 1.23)	.92	–1.30 (–1.55, –1.05)	.08
Quartiles 2–4	21.1 (21.0, 21.3)	.14	0.94 (0.79, 1.13)	.52	–1.45 (–1.59, –1.30)	.24
Dehydroepiandrosterone						
Quartile 1	20.8 (20.6, 21.1)		1.00		–1.82 (–2.11, –1.55)	
Quartile 2	20.9 (20.6, 21.1)	.88	1.10 (0.89, 1.35)	.39	–1.53 (–1.80, –1.27)	.13
Quartile 3	21.3 (21.0, 21.6)	.03	0.93 (0.75, 1.15)	.49	–1.28 (–1.53, –1.03)	.004
Quartile 4	21.3 (21.0, 21.6)	.02	0.88 (0.70, 1.09)	.24	–1.38 (–1.62, –1.13)	.02
Quartiles 2–4	21.2 (21.0, 21.3)	.05	0.97 (0.81, 1.15)	.70	–1.39 (–1.54, –1.25)	.007

*Notes*: CI = confidence interval.

^*^Adjusted for age, body mass index, alcohol, smoking, diabetes, and chronic kidney disease; *p* for difference versus Quartile 1.

^†^Adjusted for age, body mass index, alcohol, smoking, diabetes, chronic kidney disease, treatment (aspirin/placebo), and baseline grip strength; *p* for difference versus Quartile 1.

Participants with testosterone and DHEA concentrations in each of Q3 and Q4, and Q2 to Q4 combined, had statistically significantly less decline in grip strength over 4 years compared with those in their respective Q1, adjusted for age, BMI, alcohol, smoking, diabetes, chronic kidney disease, treatment allocation, and the respective baseline physical function measure (Table 2). For testosterone concentrations in Q2 to Q4 combined, the mean decline in grip strength was –1.39 kg (95% CI –1.54 to –1.24) versus Q1 –1.75 kg (–2.00 to –1.50). DHEA concentrations in Q2 to Q4 combined were associated with less decline in grip strength compared with those in Q1 (mean –1.39 kg, –1.54 to –1.25 vs –1.82 kg, –2.11 to –1.55). The impact of the change in hormone concentrations after 3 years on the association between baseline hormones and change in grip strength over 4 years was then examined in 392 participants with repeat hormone measures. With additional adjustment for the change in each hormone concentration over 3 years (Supplementary Table 2), participants with testosterone concentrations in Q4 had statistically significantly less decline in grip strength (mean –0.70 kg, 95% CI –1.43 to 0.02) compared with those in Q1 (mean –2.17 kg, –2.89 to –1.45). In an exploratory analysis, no significant associations were observed between the change in each of the sex hormone concentrations from baseline to 3 years and the change in grip strength over 4 years (Supplementary Table 3).

In adjusted analysis, baseline mean PCS scores were higher for participants in each quartile above Q1 compared with those in Q1 for testosterone (Q2 to Q4 combined *p* < .001), DHEA (*p* < .001), and estrone (*p* < .001; Table 3). The likelihood of having PCS scores classified as “poor” was lower in participants with higher concentrations (Q2 to Q4 combined vs Q1) of testosterone (odds ratio [OR] 0.86, 95% CI 0.76–0.98), DHEA (OR 0.81, 95% CI 0.71–0.92), and estrone (OR 0.82, 95% CI 0.72–0.94). In the longitudinal analysis, baseline DHEA concentrations in the top 50% were associated with a statistically significant lower likelihood of a decline in PCS score (Q3 vs Q1 *p* = .05; Q4 vs Q1 *p* = .002). No significant associations were observed for testosterone or estrone. With additional adjustment for the change in each hormone concentration over 3 years, baseline hormone concentrations were not significantly associated with the change in PCS scores (Supplementary Table 2). The changes in sex hormones over 3 years and the change in PCS scores were not associated (Supplementary Table 3).

**Table 3.** Association Between Baseline Plasma Concentrations of Sex Hormones and Self-Reported Physical Function at Baseline (*n* = 5 447) and Its Change Over 4 Years (*n* = 3 113)

	PCS Score at Baseline^*^		Poor PCS Score at Baseline^*^		Change in PCS Score^†^	
	Mean (95% CI)	*p* Value	Odds Ratio (95% CI)	*p* Value	Mean (95% CI)	*p* Value
Testosterone						
Quartile 1	47.2 (46.8, 47.6)		1.00		–1.62 (–2.16, –1.08)	
Quartile 2	47.9 (47.5, 48.4)	.02	0.89 (0.77, 1.04)	.16	–2.08 (–2.62, –1.53)	.24
Quartile 3	48.2 (47.7, 48.7)	.002	0.85 (0.72, 1.00)	.053	–1.58 (–2.17, –0.98)	.91
Quartile 4	48.4 (47.9, 48.9)	<.001	0.83 (0.71, 0.98)	.02	–1.36 (–1.93, –0.80)	.52
Quartiles 2–4	48.2 (47.9, 48.4)	<.001	0.86 (0.76, 0.98)	.02	–1.69 (–2.01, –1.36)	.84
Estrone						
Quartile 1	47.0 (46.5, 47.4)		1.00		–2.06 (–2.67, –1.45)	
Quartile 2	47.8 (47.3, 48.2)	.01	0.89 (0.76, 1.05)	.16	–1.72 (–2.27, –1.17)	.42
Quartile 3	48.3 (47.8, 48.7)	<.001	0.79 (0.67, 0.93)	.004	–1.45 (–2.00, –0.89)	.15
Quartile 4	48.5 (48.1, 49.0)	<.001	0.78 (0.66, 0.92)	.003	–1.52 (–2.07, –0.97)	.21
Quartiles 2–4	48.2 (47.9, 48.4)	<.001	0.82 (0.72, 0.94)	.003	–1.56 (–1.88, –1.25)	.16
Dehydroepiandrosterone						
Quartile 1	47.0 (46.5, 47.4)		1.00		–2.33 (–2.93, –1.72)	
Quartile 2	47.8 (47.4, 48.3)	.01	0.87 (0.74, 1.02)	.08	–1.94 (–2.51, –1.37)	.36
Quartile 3	48.3 (47.9, 48.8)	<.001	0.77 (0.66, 0.91)	.002	–1.52 (–2.06, –0.98)	.05
Quartile 4	48.4 (48.0, 48.9)	<.001	0.80 (0.68, 0.94)	.006	–1.05 (–1.59, –0.52)	.002
Quartiles 2–4	48.2 (47.9, 48.5)	<.001	0.81 (0.71, 0.92)	.002	–1.49 (–1.80, –1.17)	.02

*Notes*: CI = confidence interval; PCS = physical component summary.

^*^Adjusted for age, body mass index, alcohol, smoking, diabetes, and chronic kidney disease; *p* for difference versus Quartile 1.

^†^Adjusted for age, body mass index, alcohol, smoking, diabetes, chronic kidney disease, treatment (aspirin/placebo), and baseline PCS score; *p* for difference versus Quartile 1.

There were no interactions between obesity status or age and hormone concentrations for their association with physical function, except for the interaction between DHEA concentration and obesity status (*p* for interaction .02), and between estrone concentration and obesity status (*p* for interaction .08), for their association with grip strength decline over 4 years. In participants with BMI ≥30 kg/m^2^, DHEA and estrone concentrations in each of Q3 and Q4, and Q2 to Q4 combined were associated with reduced decline in grip strength, which was not observed in participants with BMI <30 kg/m^2^ (Supplementary Table 4).

## Discussion

In our cohort of initially healthy, community-dwelling women aged 70 years and older, concentrations of testosterone and DHEA above the 50th centile were associated with less decline in grip strength over 4 years. Higher DHEA was also associated with a less likely decline in overall self-reported physical function.

The interpretation of our findings for testosterone is somewhat challenging. Whereas DHEA continues to decline with increasing age in older women, testosterone tends to increase with age from the seventh to eighth decade (2,4,5). Testosterone exerts anabolic actions in musculoskeletal tissue, mediated directly through androgen receptors and indirectly by aromatization to estradiol (33,34). In our study, the association between higher baseline testosterone and less decline in grip strength over 4 years persisted in the exploratory analysis with additional adjustment for the change in testosterone concentrations from baseline to 3 years. This suggests that high concentrations of endogenous testosterone predict less future decline in muscle strength in older women, independent of the gradual increase in testosterone concentrations. The only prior prospective cohort study with which to compare our findings reported an association between low free testosterone measured by immunoassay and a decrease in grip strength over 2 years in community-dwelling women aged 70–84 years (35). Not only do, immunoassays provide inaccurate estimates of free testosterone (36), but the clinical significance of free testosterone remains uncertain (37,38). Our systematic review and meta-analysis revealed 10 studies of testosterone and muscle function/strength, most of which were in midlife and older women, with only one of women aged less than 40 years (6). The latter measured testosterone by LCMS and found no association between testosterone and muscle strength (39). Our systematic review and meta-analysis only identified 2 randomized controlled trials of exogenous testosterone on muscle strength and 3 on muscle mass in postmenopausal women, neither of which showed a significant treatment effect (40). These trials were limited by small sample size (<20 participants per group) and short treatment durations (6–16 weeks). Given the anabolic actions of testosterone in muscle, randomized controlled trials with larger sample sizes and longer treatment durations are needed to determine whether exogenous testosterone has favorable effects on physical function in older postmenopausal women.

Studies with which to compare our findings for DHEA are limited. Two prospective cohort studies reported an association between lower blood concentrations of DHEAS and a decline in grip strength in community-dwelling women aged ≥65 years (8,9). One cross-sectional study showed a positive relationship between DHEAS/DHEA concentrations and grip strength in women aged ≤49 years, aged 50–64 years, and those aged ≥65 years, with strongest association observed in women aged ≤49 years (41). DHEAS is almost exclusively produced by the adrenals and provides a circulating reservoir for DHEA (42). DHEA, in turn, serves as the main precursor for testosterone and estrogen production in postmenopausal women (42). DHEAS has been traditionally used as an index of adrenal pre-androgen production due to the availability of immunoassays for its measurement. However, unconjugated DHEA, now measurable by mass spectrometry-based methods, is potentially a better marker of pre-androgen effects due to its much more rapid clearance (43). DHEA declines significantly, linearly with age (2,44). The greater decline in both grip strength and self-reported physical function over 4 years in participants with the lowest DHEA concentrations in our study suggests that DHEA is a clinically meaningful biomarker of biological aging and general health, particularly in participants with obesity. It is noteworthy that a systematic review demonstrated inconsistent effects of DHEA supplementation on muscle strength and physical function in randomized clinical trials of older adults (45). Consequently, further studies are needed to determine the effects of exogenous DHEA on measures of physical function in community-based older women.

Estrogens may contribute to the maintenance of skeletal muscle function (46). Mice lacking the estrogen alpha receptor exhibit early skeletal muscle fatigue and reduced skeletal muscle glucose disposal (47). Estrogen insufficiency has been implicated as contributing to skeletal muscle apoptosis (48). However, our findings for estrone were limited to a positive association with the baseline physical health-related quality of life, and an association between concentrations above the 50th centile and less grip strength decline over 4 years in participants with obesity. A systematic review and meta-analysis found no association between postmenopausal estrogen therapy and lean muscle mass in randomized clinical trials (49). Further studies of muscle function as a primary outcome are needed to determine whether estrogen therapy protects against postmenopausal muscle loss.

Our study has several strengths. It included over 5 000 initially healthy community-dwelling women aged 70–95 years, free of chronic disability at enrollment, drawn from a large, well-characterized, community-based clinical trial. Physical function was assessed using standard, validated, well-described instruments and performed by trained observers according to the published protocols to minimize measurement bias. Findings were consistent when baseline physical functions were examined in complementary ways, as both continuous variables and dichotomous variables defined using clinically meaningful cutoffs. Sex hormones were measured using LCMS, which provides precision at low blood concentrations.

Our study had some limitations. A major limitation was the 44% loss to follow-up which might introduce selection bias. However, the differences between participants with follow-up data and those without were marginal other than the follow-up participants having a lower likelihood of diabetes and greater PCS score at baseline, making it less likely that the loss to follow-up would have affected our results. The assessment of hormone changes from baseline to 3 years (ie, exposure) preceded the assessment of physical function changes from baseline to 4 years (ie, outcome), suggesting a potential temporal relationship between hormone changes and functional changes. However, the full trajectory of change in the hormone variables is not known, it is possible that an additional year of aging would result in further change in sex hormone levels, which might cause PCS scores to be lower at 4 years than if they had been measured at 3 years. Being observational in nature, our findings provide no information as to whether there is a causative relationship between DHEA and testosterone and decline in physical function, and there remains the possibility of residual confounding. The ASPREE study did not measure muscle mass. Despite being one of the most commonly used assessments of muscle performance, grip strength may not be sufficiently sensitive to subtle changes in muscle strength and performance within the normal range of physical function in relatively healthy older women. Muscle power has been identified as a better measure of muscle function as it declines earlier and faster than muscle mass and strength in healthy older individuals (50). Our study included volunteers in a long-term clinical trial who may have had a greater interest in their health, which might influence the generalizability of our findings. Participants were primarily of European ancestry, and thus our findings may not be generalizable to other populations.

In conclusion, our study demonstrated higher endogenous concentrations of testosterone and DHEA indicate a lower likelihood of physical function decline in community-based women aged 70 years and over. Further studies are warranted to determine the effects of testosterone and DHEA therapy on sensitive measures of muscle strength and performance in older women.

## Supplementary Material

glad287_suppl_Supplementary_Tables_1-4
