# Supplementary material for: Associations Between Blood Concentrations of Sex Hormones and Physical Function in Community-Dwelling Older Women: A Prospective Cohort Study
Source: J Gerontol A Biol Sci Med Sci. 2023 Dec 29;79(4):glad287. doi: 10.1093/gerona/glad287 (PMC10919882; doi:10.1093/gerona/glad287)
Supplement: glad287_suppl_Supplementary_Tables_1-4 [file glad287_suppl_supplementary_tables_1-4.pdf]

Table of contents

Associations between blood concentrations of sex hormones and physical function in community-dwelling older women: a prospective cohort study

eTable 1. Characteristics of participants in those who completed 4-year follow-up and those who did not

eTable 2. Association between plasma concentrations of sex hormones at baseline and change in physical function over 4 years (n=392), adjusted for change in plasma concentrations of sex hormones over 3 years

eTable 3. Association between change in plasma concentrations of sex hormones at 3 years and change in physical function over 4 years (n=392)

eTable 4. Association between plasma concentrations of sex hormones at baseline and change in grip strength over 4 years, stratifies by obesity status

eTable 1. Characteristics of participants in those who completed 4-year follow-up and those who did not

	Completed N=3113	Not completed N=2334	p-value*
Age, years	74.1 (71.9-77.5)	73.7 (71.5-77.8)	0.02
Body mass index, kg/m^2	28.0 (5.0)	28.2 (5.1)	0.047
Current/former smoker, no (%)	1029 (33.1)	837 (35.9)	0.03
Current/former alcohol user, no (%)	2434 (78.2)	1846 (78.7)	0.42
Aspirin group, no (%)	1559 (50.1)	1167 (50.0)	0.95
Diabetes, no (%)	214 (6.9)	218 (9.3)	0.001
Chronic kidney disease, no (%)	563 (18.5)	427 (18.9)	0.72
Grip strength, kg	21.0 (5.3)	21.2 (5.4)	0.40
SF-12 PCS score (0-100)	48.3 (8.8)	47.3 (9.5)	<0.0001

Data are presented as median (interquartile range), mean (SD), or no (%)
*P for difference between the two groups using Mann-Whitney U test, independent samples t test, or chi square test, when appropriate

eTable 2. Association between plasma concentrations of sex hormones at baseline and change in physical function over 4 years (n=392), adjusted for change in plasma concentrations of sex hormones over 3 years

	Change in grip strength (kg)*		Change in SF-12 PCS score*	
	Mean (95% CI)	p-value	Mean (95% CI)	p-value
**Testosterone**				
Quartile 1	-2.17 (-2.89, -1.45)		-1.69 (-3.37, -0.01)	
Quartile 2	-1.42 (-2.16, -0.69)	0.16	-2.39 (-4.05, -0.73)	0.56
Quartile 3	-2.24 (-2.97, -1.51)	0.89	-1.30 (-3.00, 0.40)	0.75
Quartile 4	-0.70 (-1.43, 0.02)	0.005	-0.85 (-2.52, 0.83)	0.49
Quartiles 2 to 4	-1.45 (-1.88, -1.03)	0.09	-1.52 (-2.49, -0.55)	0.87
**Oestrone**				
Quartile 1	-2.16 (-2.90, -1.41)		-2.71 (-4.40, -1.02)	
Quartile 2	-1.79 (-2.55, -1.03)	0.50	-1.62 (-3.34, 0.10)	0.37
Quartile 3	-1.36 (-2.10, -0.62)	0.14	-0.36 (-2.03, 1.31)	0.054
Quartile 4	-1.23 (-2.00, -0.45)	0.11	-1.48 (-3.26, 0.30)	0.35
Quartiles 2 to 4	-1.46 (-1.89, -1.03)	0.14	-1.14 (-2.12, -0.15)	0.12
**Dehydroepiandrosterone**				
Quartile 1	-2.02 (-2.76, -1.28)		-1.87 (-3.57, -0.17)	
Quartile 2	-1.65 (-2.38, -0.91)	0.48	-2.27 (-3.92, -0.62)	0.74
Quartile 3	-1.91 (-2.66, -1.16)	0.84	-2.69 (-4.39, -0.99)	0.50
Quartile 4	-0.95 (-1.76, -0.13)	0.07	0.67 (-1.18, 2.51)	0.06
Quartiles 2 to 4	-1.52 (-1.95, -1.09)	0.32	-1.53 (-2.50, -0.55)	0.89

CI: confidence interval; PCS: physical component summary
*adjusted for age, body mass index, alcohol, smoking, diabetes, chronic kidney disease, treatment (aspirin/placebo), respective baseline physical function measure, and change in plasma concentrations of sex hormones; p for difference versus quartile 1

eTable 3. Association between change in plasma concentrations of sex hormones at 3 years and change in physical function over 4 years (n=392)

	Change in grip strength (kg)*		Change in SF-12 PCS score*	
	Mean (95% CI)	p-value	Mean (95% CI)	p-value
**Change in testosterone**				
Quartile 1, median -0.10 (IDR -0.31, -0.07) nmol/L	-1.86 (-2.61, -1.11)		-2.98 (-4.70, -1.26)	
Quartile 2, 0 (-0.03, 0) nmol/L	-1.47 (-2.15, -0.80)	0.47	-0.27 (-1.81, 1.27)	0.02
Quartile 3, 0.03 (0.03, 0.07) nmol/L	-1.44 (-2.25, -0.62)	0.47	-2.49 (-4.35, -0.62)	0.71
Quartile 4, 0.21 (0.10, 0.66) nmol/L	-1.79 (-2.54, -1.04)	0.90	-0.94 (-2.65, 0.78)	0.10
**Change in oestrone**				
Quartile 1, -79.52 (-203.42, -48.08) pmol/L	-1.57 (-2.34, -0.79)		-1.57 (-3.31, 0.17)	
Quartile 2, -14.79 (-33.29, 0) pmol/L	-1.60 (-2.31, -0.88)	0.96	-0.31 (-1.94, 1.32)	0.31
Quartile 3, 25.89 (11.10, 51.78) pmol/L	-1.57 (-2.32, -0.82)	1.00	-1.20 (-2.88, 0.47)	0.77
Quartile 4, 107.26 (66.57, 236.71) pmol/L	-1.83 (-2.59, -1.07)	0.64	-3.27 (-4.97, -1.57)	0.17
**Change in dehydroepiandrosterone**				
Quartile 1, -1.15 (-2.08, -0.62) nmol/L	-2.07 (-2.86, -1.28)		-2.87 (-4.67, -1.07)	
Quartile 2, -0.29 (-0.52, -0.10) nmol/L	-1.69 (-2.43, -0.94)	0.49	-0.37 (-2.06, 1.33)	0.048
Quartile 3, 0.21 (0, 0.42) nmol/L	-1.12 (-1.86, -0.37)	0.10	-1.86 (-3.56, -0.17)	0.44
Quartile 4, 0.83 (0.52, 2.15) nmol/L	-1.70 (-2.45, -0.95)	0.52	-1.12 (-2.82, 0.56)	0.18

IDR: interdecile range; CI: confidence interval; PCS: physical component summary
*adjusted for age, body mass index, alcohol, smoking, diabetes, chronic kidney disease, treatment (aspirin/placebo), and respective baseline sex hormone and physical function measure; p for difference versus quartile 1

eTable 4. Association between plasma concentrations of sex hormones at baseline and change in grip strength over 4 years, stratifies by obesity status

	Body mass index <30 kg/m^2		Body mass index $\geq$30 kg/m^2	
	Mean (95% CI) *	p-value*	Mean (95% CI) *	p-value*
**Testosterone**				
Quartile 1	-1.64 (-1.94, -1.34)		-2.00 (-2.45, -1.55)	
Quartile 2	-1.65 (-1.95, -1.35)	0.97	-1.78 (-2.25, -1.32)	0.51
Quartile 3	-1.08 (-1.41, -0.75)	0.01	-1.86 (-2.35, -1.37)	0.68
Quartile 4	-1.03 (-1.34, -0.72)	0.006	-1.40 (-1.88, -0.91)	0.07
Quartiles 2 to 4	-1.27 (-1.45, -1.09)	0.04	-1.68 (-1.96, -1.40)	0.24
**Oestrone**				
Quartile 1	-1.39 (-1.71, -1.07)		-2.44 (-3.05, -1.83)	
Quartile 2	-1.44 (-1.73, -1.15)	0.82	-2.00 (-2.52, -1.49)	0.28
Quartile 3	-1.35 (-1.65, -1.04)	0.85	-1.68 (-2.14, -1.22)	0.05
Quartile 4	-1.28 (-1.61, -0.95)	0.64	-1.43 (-1.82, -1.05)	0.006
Quartiles 2 to 4	-1.36 (-1.54, -1.18)	0.89	-1.65 (-1.90, -1.40)	0.02
**Dehydroepiandrosterone**				
Quartile 1	-1.51 (-1.84, -1.17)		-2.56 (-3.06, -2.06)	
Quartile 2	-1.38 (-1.69, -1.07)	0.58	-1.89 (-2.38, -1.41)	0.06
Quartile 3	-1.34 (-1.64, -1.04)	0.46	-1.19 (-1.64, -0.75)	<0.001
Quartile 4	-1.28 (-1.58, -0.98)	0.32	-1.61 (-2.06, -1.16)	0.006
Quartiles 2 to 4	-1.33 (-1.50, -1.15)	0.36	-1.55 (-1.81, -1.28)	<0.001

CI: confidence interval

*adjusted for age, body mass index, alcohol, smoking, diabetes, chronic kidney disease, treatment (aspirin/placebo), and baseline grip strength; p for difference versus quartile 1
